# Phase-Transition Nanodroplets for Real-Time Photoacoustic/Ultrasound Dual-Modality Imaging and Photothermal Therapy of Sentinel Lymph Node in Breast Cancer

**DOI:** 10.1038/srep45213

**Published:** 2017-03-24

**Authors:** Lu Yang, Juan Cheng, Yuli Chen, Shengjie Yu, Fengqiu Liu, Yang Sun, Yu Chen, Haitao Ran

**Affiliations:** 1 Department of Ultrasound, Second Affiliated Hospital of Chongqing Medical University & Chongqing Key Laboratory of Ultrasound Molecular Imaging, Chongqing, 400010, China; 2 Department of Breast, Thyriod, Pancreas Surgery, Second Affiliated Hospital of Chongqing Medical University, Chongqing 400010, China; 3 Department of Urinary Surgery, Second Affiliated Hospital of Chongqing Medical University, Chongqing 400010, China; 4 State Key Laboratory of High Performance Ceramics and Superfine Microstructure, Shanghai Institute of Ceramics, Chinese Academy of Sciences, Shanghai, 200050, P. R. China

## Abstract

Pathological status of lymph nodes (LNs) plays a critical role in staging and treatment for the patients with breast cancer. Sentinel lymph node biopsy has become the standard method in determining pathological status of axillary LNs. Therefore, the determination of sentinel lymph nodes (SLNs) and therapy of metastatic LNs are highly desirable in clinic. Herein, an unprecedented carbon nanoparticles (CNs)-incorporated liquid-gas phase-transition nanodroplets (CNPs) with strong near-infrared (NIR) absorption, good biocompatibility, excellent photoacoustic (PA) and ultrasound (US) contrast, and high photothermal-conversion efficiency are reported in this study. Upon laser irradiation, liquid-gas phase transition of the CNPs has been demonstrated to provide excellent contrasts for PA/US dual-modality imaging both *in vitro* and *in vivo*. Additionally, the CNPs are capable of staining lymph nodes, which can contribute significantly to the identification of LNs with naked eyes. With increased laser energy, the CNPs exhibit the high performance in killing the breast cancer cells both *in vitro* and *in vivo*, due to the photothermal effect induced from the CNs within CNPs. These results suggest that the developed multifunctional phase-transition nanodroplets have high potential to act as the theranostic agents in both SLNs detection and therapy of metastatic LNs.

Nano-based particles, droplets, and capsules have been extensively employed in versatile applications including primary tumor diagnosis and therapy over the past decades^1^,^2^,^3^,^4^,^5^. However, in addition to the blood vessels, these agents are also the potential tracers of the lymphatic duct, offering a promising method in the diagnosis of the metastasis of the tumor. Breast cancer is one of the most common cancers in females. The presence or absence of axillary lymph nodes (LNs) metastasis remains the most powerful prognostic feature when staging breast cancer and guiding the treatment. Traditionally, lymph node status is established by the dissection of axillary lymph nodes, but this approach has serious complications such as upper limb lymphedema, motor and sensory disturbances in pathologic node-negative patients. Currently, sentinel lymph node biopsy (SLNB) has been widely performed because it avoids above-mentioned complications by completed dissection of axillary lymph nodes^6^,^7^.

The SLN is defined as the first regional lymph node or a group of nodes that receives lymphatic drainage from a primary malignant tumor. Lymphatic mapping with SLN permits staging of malignant tumors in an effort to avoid complete nodal dissection and its associated morbidity. The introduction of SLNB has therefore revolutionized the surgical management of breast cancer, providing a minimally invasive means to detect early and subclinical metastasis and for early therapeutic intervention. Hence, the development of effective method to detect and adequately diagnose SLN with good sensitivity and specificity is a definite and urgent clinical need for breast cancer treatment. Currently, SLN is clinically mapped with blue dye and/or radio-labeled sulfur colloid^8^,^9^. It has been reported that SLNB identification rates is between 90 to 95%, and the sensitivity of SLNB is between 88 to 95% in experienced hands^10^,^11^,^12^. However, we should still notice some practical limitations of above methods. First, the procedure lacks real-time imaging guidance. Second, 5%–10% false negative rate has been also reported. Third, side effects caused by blue dye such as allergy, skin and subcutaneous tissue necrosis in injection site, and radiation exposure caused by radio-labeled sulfur colloid cannot be avoided^13^,^14^. Finally, SLNB is often performed as a staged procedure, requiring that breast cancer patients undergo two or more operations for mapping the lymph nodes of axilla. Therefore, it is an unmet challenge for an imaging technique that is widely available and noninvasive to detect and adequately diagnose SLN in real-time.

A large number of imaging models have been tested in animals and patients in attempts to improve the accuracy and safety of SLN biopsy^15^,^16^,^17^,^18^. Lymphatic mapping with contrast-enhanced ultrasonography (CEUS) has shown the advantages of real-time imaging in lymphatic vessel and SLN. The utilization of CEUS can clearly describe the SLN number, size and shape. Several reports suggest that axillary CEUS is a potential and valuable technique for identifying axillary lymphatic metastasis^19^,^20^. However, it has the limitations of non-staining in SLN and shorter retention time for ultrasonic contrast after the introduction of microbubbles, which is critical to determine the possibility of using intraoperative ultrasound for localization of SLN during resection. Previously, we have successfully introduced CEUS into the LNs detection by employing the Sudan black-incorporated poly(lactide-co-glycolide acid) (PLGA) microbubbles^21^. The prepared PLGA microbubbles could map and stain LNs. The microbubbles continuously accumulated into SLNs, which could last for long duration *in vivo* to enhance ultrasound imaging. However, the efficacy of a single-imaging modality remains to be further explored and improved. Photoacoustic (PA) imaging is a hybrid biomedical imaging modality that possesses the contrast superiority of optical imaging and the resolution superiority/penetration ability of ultrasound imaging^22^. PA imaging has already been used for mapping SLNs in recent years, but LNs have poor ability in absorbing light, therefore the exogenous contrast agent should be employed. These agents include some dyes, metal nanoparticles and fluorescent agents, such as india ink, methylene blue, gold nanorods, indocyanine green, etc.^4^,^23^,^24^,^25^,^26^,^27^. As mentioned above, the ideal contrast agents for lymphatic mapping should have several desirable characteristics: first, it should be promptly absorbed by the lymphatics and quickly transported to the first-echelon nodes; second, it should have sustained-accumulation capability in the SLNs at high amounts, and provide good contrast to background counts in the nodal basin; third, it should have minimal pass-through effect to non-SLN nodes.

The duration of CEUS is short with the assistance of contrast agent used in clinic. Comparatively, the PA imaging can offer quite long duration with proper contrast agent, which is critical to help surgeon to detect and locate SLNs. However, the penetration depth of PA imaging is lower than that of CEUS. In order to improve the detection rate and accuracy rate of SLNs, the introduction of multimodal imaging is typically considered. Especially, the combination of CEUS and PA imaging has the simultaneously advantages of real-time dynamic detection, high penetrability and long duration. These advantages are expected to help surgeon to accurately detect and locate SLNs for biopsy. In addition, the size of commonly used ultrasound microbubbles in clinic is in micrometer-size range. Their stability is poor and the imaging duration is short. These disadvantages limit their application in the lymph nodes diagnosis. The design of phase-transition nano-droplets can potentially solve this critical issue. The phase-transitional nano-droplets can enter the tumor tissues and cells because of their small nano-sizes. The post phase transition by external irradiation can effectively respond to ultrasound and provide the excellent contrast-enhanced US imaging, which is controllable, stable and continuous.

Carbon nanoparticles (CNs) represent a new potential LNs tracer. They have no biological activity and immunogenicity, which have been approved by Chinese Food and Drug Administration for clinical application^28^,^29^,^30^,^31^. They have the main effective component of nanoscale-activated carbon and have excellent NIR-absorption property, which makes them a potential PA-imaging contrast agent. Herein, we introduce, for the first time, a new dual-modality contrast agent for mapping SLN by using US and PA imaging with CNs. The typical carbon nanoparticles-incorporated nanodroplets (CNPs) have been designed and fabricated in this work, which consist of a droplet of liquid perfluorohexane (PFH) and CNs within PLGA shell. PLGA is FDA approved and has been clinically used in biological and medical applications. PFH with the boiling point of 56 °C is a stable liquid at room temperature, which can be triggered to gaseous phase with proper heat energy. Upon illumination with proper laser energy, CNs in the CNPs can absorb light and transform the light energy into heat to provoke PFH. The liquid-gas phase transition of CNPs has been successfully demonstrated both *in vitro* and *in vivo*, providing excellent contrast for both PA and US imaging. Moreover, the CNPs have photothermal-conversion effect after exposure to laser. Photothermal therapy is highly effective in oncotherapy^4^,^32^,^33^. In this study, we have shown that CNPs has the therapeutic effect on the tumor, as demonstrated both *in vitro* and *in vivo*. Therefore, the developed CNPs are the promising multifunctional theranostic agent in both SLN detection and therapy of metastatic LN.

## Results

### Synthesis and characterization of CNPs

**Figure 1:**
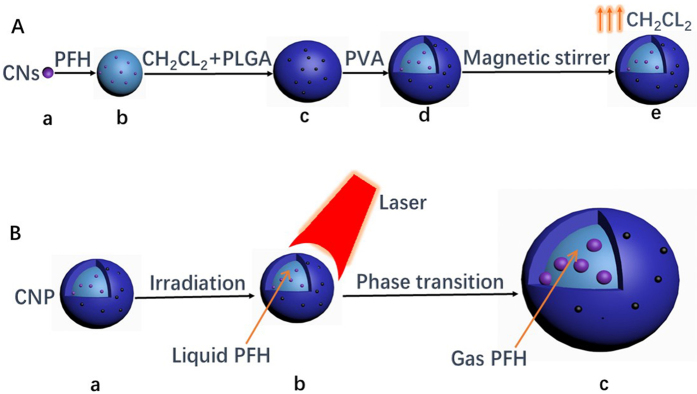
(**A)**, schematic diagram of the fabrication process for carbon nanoparticles nanodroplets (CNPs) (a–e). (**B**). perfluorohexane (PFH) liquid-gas transition after laser irradiation (a–c).

Figure 1A(a–e) shows the fabrication process of CNPs: (a-b) PFH was embedded with CNs, which was suspended in water as the first emulsion; (b-c) PFH and CNs were encapsulated by PLGA dissolved in CH_2_Cl_2_ as the second emulsion; (c-d) PFH, CNs and PLGA were encapsulated by PVA as the third emulsion; (d-e) CH_2_Cl_2_ on PLGA shell evaporated by magnetic stirring. Fig. 1B(a–c) PFH liquid-gas phase transition occurred after the exposure of CNPs to proper laser energy.

**Figure 2:**
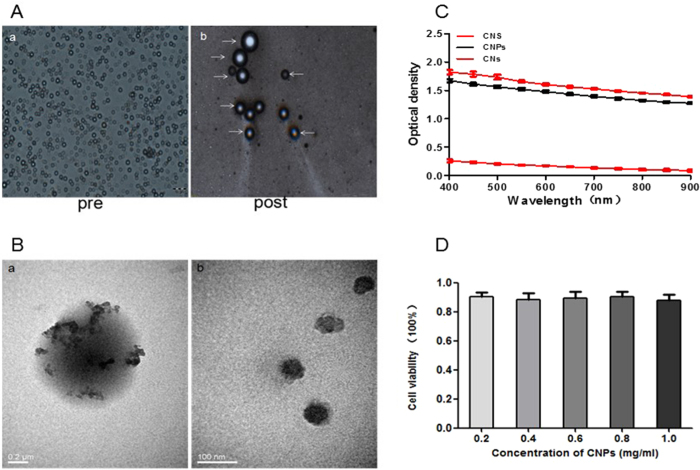
(**A)** (a and b), optical microscopic images of the CNPs before and after laser irradiation. The white arrows in (b) indicate the phase transition of CNPs. (**B)**, (a), transmission electron microscope (TEM) images of a CNP. Black granular carbon nanoparticles (CNs) and gray black PFH were encapsulated in the white poly(lactide-co-glycolide acid) (PLGA) shell; (b), TEM images of CNs. (**C**), the optical density of CNPs, pure CNs and NPs, as measured by spectrophotometry. (**D**), cytotoxicity results of CNPs on RAW264.7 cells (mean ± standard deviation for different concentrations of CNPs).

Figure 2A shows the size and morphology of CNPs under optical microscope (Olympus CKX41, CANADA) before and after laser irradiation. It is shown that the CNPs exhibited uniform size and high monodispersity with spherical morphology before laser irradiation. After laser irradiation, the volume of parts of CNPs obviously increased due to the PFH liquid-gas phase transition. To learn more about the internal structure of CNPs, TEM images at high-magnification were acquired. Fig. 2B shows the structure of a single CNP and CNs under TEM observation. The TEM images exhibit that the internal structure of CNPs was clearly visible. The black granular CNs (Fig. 2Bb) were inserted into gray black PFH. Finally, they were all encapsulated in the white PLGA shell (Fig. 2Ba). The structure of CNPs shown in TEM image was consistent with the schematic diagram (Fig. 1). In addition, CNPs exhibited a narrow size distribution with an average hydrodynamic diameter of 435.9 ± 41.31 nm (mean ± standard deviation) (Supplementary Fig. S1A, S1B and S1C). Such a size was far smaller than that of clinical ultrasonic microbubble used for SLN detection. Therefore, CNPs can be easily and promptly absorbed by the lymphatics and quickly transport to SLN after subcutaneous injection. The average zeta potential was determined to be −25.5 ± 0.39 mV (mean ± standard deviation) (Supplementary Fig. S1D, S1E and S1F). Moreover, the introduction of CNs in CNPs could endow the nanodroplets with the property of optical absorption. As shown in Fig. 2C, the absorption spectra of CNPs and pure CNs were similar to each other, but the absorption of NPs was negligible. These results implied that CNPs inherited the optical absorption properties of CNs without significant alteration during synthetic process. In addition, the CNPs exhibited smooth and powerful optical density at the wavelength range of 400–900 nm. Such an optical property has been shown critical to the successful application in PA imaging and photothermal therapy.

### Cytotoxicity

After RAW264.7 cells were incubated with CNPs at concentrations of 0.2, 0.4, 0.6, 0.8 and 1.0 mg/mL, the cell viabilities were determined to be 90.1%, 88.5%, 89.2%, 90.5%, and 87.6%, respectively (Fig. 2D). These results neither showed a definite correlation between CNPs concentration and cell viability, nor did they show a significant difference with the control groups (P > 0.05), suggesting that CNPs have low cytotoxicity toward RAW264.7 cell lines.

### *In Vitro* phagocytosis study

**Figure 3:**
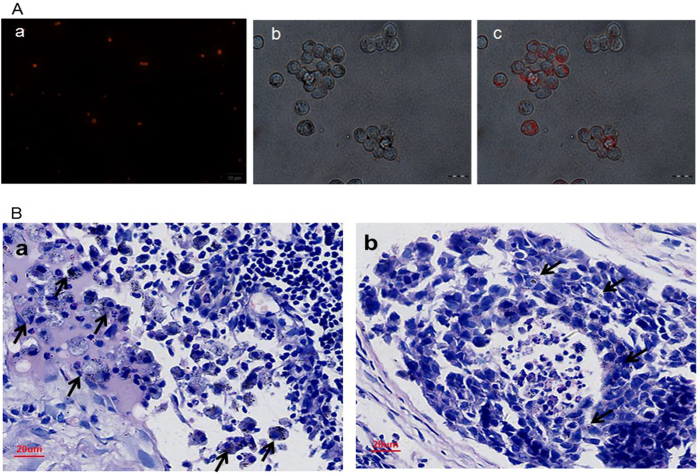
(**A)**, (a), fluorescence microscope image of Dil-labeled CNPs. (b and c), optical microscope and fluorescence microscope image of CNPs phagocytosed by macrophages, respectively. (**B)**, (a), hematoxylin-eosin (HE) staining of metastatic LN tissues after CNPs injection for 48 h. The black arrows indicate the macrophages phagocytized CNPs. (b), HE staining of metastatic LN tissues after CNPs injection for 48 h. The black arrows indicate the CNPs were around the tumor cells.

DiI-labeled CNPs were successfully synthesized and observed in the fluorescence microscope image (Fig. 3Aa). A significant amount of DiI-labeled CNPs were phagocytosed by macrophages as observed by fluorescence microscopy after 3 h incubation (Fig. 3Ab,Ac). These results demonstrated that CNPs were phagocytosed and resided inside the macrophages, which suggested that CNPs could be taken into LNs by macrophages. This was an important path that the CNPs entered into LNs like other lymph tracer^21^,^34^.

### *In vivo* phagocytosis and inflammation study

A large number of macrophage-phagocytized CNPs are shown in Fig. 3Ba, which further suggests that CNPs could be taken into LNs by macrophages. These CNPs around the tumor cells are shown in Fig. 3Bb. It has been found that there is no obvious neutrophil infiltration in both of the two images, which indicates that no obvious acute inflammatory reaction occurred after CNPs entering into LNs.

### *In vitro* dual-mode imaging of CNPs

**Figure 4:**
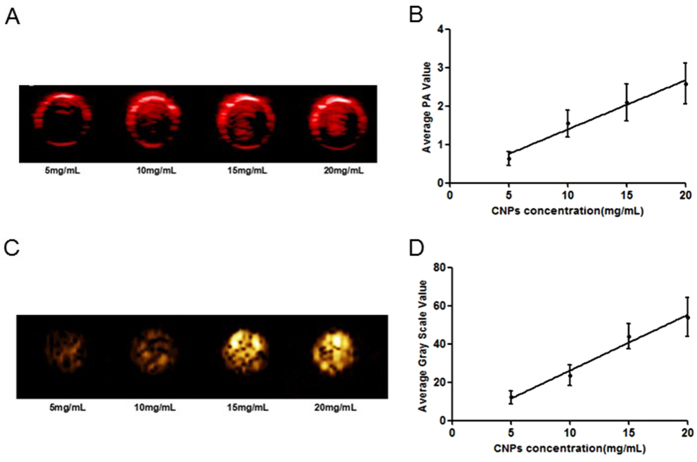
(**A**), photoacoustic (PA) images of 5, 10, 15 and 20 mg/mL CNPs in 0.2% agarose gel after irradiation. (**B**), the quantitative analysis of the PA signal intensity of concentrations in (**A**). (**C**), contrast-enhanced ultrasonography (CEUS) images of 5, 10, 15 and 20 mg/mL CNPs in 0.2% agarose gel after irradiation (808 nm, 1 W/cm^2^) for 10 s. (**D**), quantitative analysis of the average gray scale in concentrations in (**C)**.

The capability of CNPs as the contrast agents for PA imaging was firstly assessed *in vitro* using Vevo LAZR PA imaging system, and the saline groups were used as control. There was no PA contrast enhancement in the images of control groups after laser irradiation. Comparatively, the PA signals gradually increased with the increase of CNPs concentrations after laser irradiation (Fig. 4A). The PA signals originated from various concentrations of CNPs were also quantitatively analyzed (Fig. 4B), and the results confirmed that the PA signals had linear correlation according to the changes of CNPs concentration where high concentration gave rise to stronger signal intensity.

Likewise, the CEUS of CNPs was investigated and the saline groups were also employed as control. There was no ultrasonic contrast enhancement in control groups after laser irradiation. However, the CNPs of various concentrations provided good ultrasonic contrast enhancement after laser irradiation as shown in Fig. 4C. This can be explained by the PFH liquid-gas phase transition in CNPs. The CNs encapsulated in CNPs have strong optical absorption in NIR range. The light energy absorbed by CNs was converted into heat energy and PFH liquid-gas phase transition was excited afterwards. The series of conversion thus enhanced the ultrasound imaging, which further implied that CNPs could have sustained accumulation in targeted tissue, and provide good ultrasound contrast to background. The ultrasonic images of ROI were quantitatively analyzed with “average gray scale” using DFY ultrasound imaging analysis software (Fig. 4D). The gray scale of ultrasound increased with increasing of CNPs concentrations. These *in vitro* results demonstrated that CNPs can be used as the contrast agents for efficient PA and US dual imaging.

### *In vivo* dual-mode imaging of CNPs

**Figure 5:**
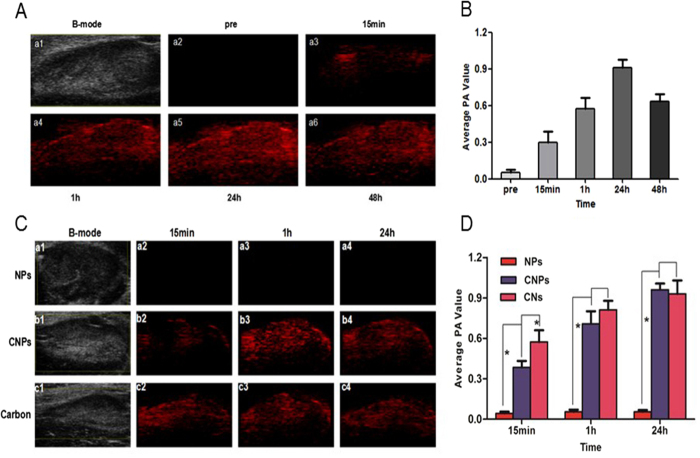
(**A**), a1, B-mode image of lymph node (LN); a2, PA-mode image of LN before CNPs injection, a3-a6, PA-mode images of LNs after CNPs injection at different time points (15 min, 1 h, 24 h, 48 h). (**B**), quantitative analysis of the PA signal intensity in **A**. (**C**). a1, b1, c1, B-mode image of LNs before agents (NPs, CNPs and CNs) injection, a2, b2, c2, PA-mode image of LNs after agents injection at 15 min, a3, b3, c3, PA-mode image of LNs after agents injection at 1 h, a4, b4, c4, PA-mode image of LNs after agents injection at 24 h. (**D**). quantitative analysis of the PA signals intensity in **C** (*p < 0.05).

We further assessed the imaging ability of CNPs as contrast agents for the *in vivo* PA and CEUS imaging using rabbits bearing VX2 tumor. The time for imaging start and the imaging duration of CNPs were critical for SLN imaging. Hence, both PA imaging and CEUS were observed and quantitatively analyzed at various time points. First, the PA imaging of popliteal fossa lymph nodes was continuously observed for 1 h and at the time points of 24 h and 48 h after CNPs injection. It was obvious at 15 min after CNPs injection. Part of the LNs showed signal enhancement in PA-mode (Fig. 5Aa3), and the whole signal of LNs was gradually enhanced after 1 h (Fig. 5Aa4). With the increasing accumulation of CNPs in LNs, the better PA-signal enhancement was acquired at 24 h (Fig. 5Aa5). Furthermore, the results showed that even at 48 h, the desirable PA imaging in LNs was also observed (Fig. 5Aa6), but a bit weaker as compared with that at 24 h, indicating that the amount of CNs was gradually reduced at this moment. Then, the PA signal of the images was quantitatively analyzed, and the results were consistent with that observed in the PA images (Fig. 5B).

Subsequently, the rabbits injected with pure CNs and NPs were employed as control groups, and the PA images were collected at 15 min, 1 h and 24 h. It was shown that the NPs group without optical absorption material had no effect in enhancing PA imaging (Fig. 5Ca2). The CNs group had the same or better effect in enhancing PA imaging especially at 15 min and 1 h, but the PA signal decreased at 2 h compared with the CNPs group (Fig. 5Cb2). Probably, the size of CNs was much smaller, therefore, they were easier and faster to arrive at LNs. PA signal of the images was also quantitatively analyzed, and the results were consistent with that observed in PA images (Fig. 5D).

**Figure 6:**
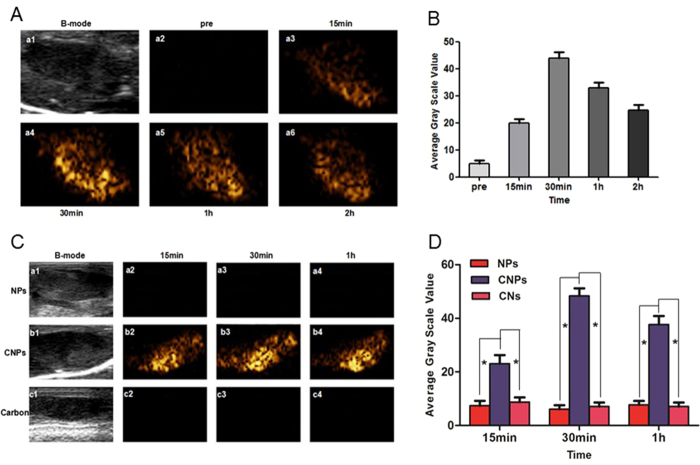
(**A**), a1, B-mode image of LNs; a2, contrast-mode image of LN before CNPs injection, a3-a6, contrast-mode images of LNs after CNPs injection (15 min, 1 h, 2 h, 24 h). (**B**), quantitative analysis of the PA signal intensity in **A. (C**). a1, b1, c1, B-mode images of LNs; a2-a4, b2-b4, c2-c4, PA-mode images of LNs after NPs, CNPs and CNs injection at different time points (15 min, 1 h and 24 h). (**D**), quantitative analysis of the PA signal intensity in **C** (*p < 0.05).

Likewise, the CEUS of popliteal fossa lymph nodes using CNPs was observed at 15 min, 30 min, 1 h and 2 h after CNPs injection and the LNs were irradiated with laser for 1 min before each observation. It was noticed that the signal of LNs began to enhance from 15 min in CEUS-mode (Fig. 6Aa3), and the best enhancement effect was obtained at about 30 min (Fig. 6Aa4). At 1 h and 2 h after injection, the effect of CEUS was still good and stable, but a bit weaker compared with that at 30 min (Fig. 6Aa5). However, after each irradiation, the CNPs in LNs were damaged after liquid-gas phase transition, which led to the poorer US enhancement at 24 h compared with PA enhancement. When the first irradiation was performed at 24 h, the effect of CEUS of LNs was also highly desirable, indicating that the CNPs were quite stable *in vivo*. In addition, the ROI of the CEUS images was quantitatively analyzed with DFY, and the result was consistent with that observed in the CEUS images (Fig. 6B).

Subsequently, the rabbits injected with pure CNs and NPs were set as control groups. The CEUS images were collected at 15 min, 30 min and 1 h. It was obviously shown that both of the control groups had no effect in CEUS (Fig. 6Ca2). ROI of the images was quantitatively analyzed with DFY, and the result was consistent with that observed in the CEUS images (Fig. 6D). The pure CNs without the material-generating gas had no effect in CEUS. However, without optical absorption material, liquid-gas phase transition did not occur, which caused no enhancement of LNs in CEUS. These results were consistent with previous reports^4^,^35^.

### LN biopsy after CNPs injection

**Figure 7:**
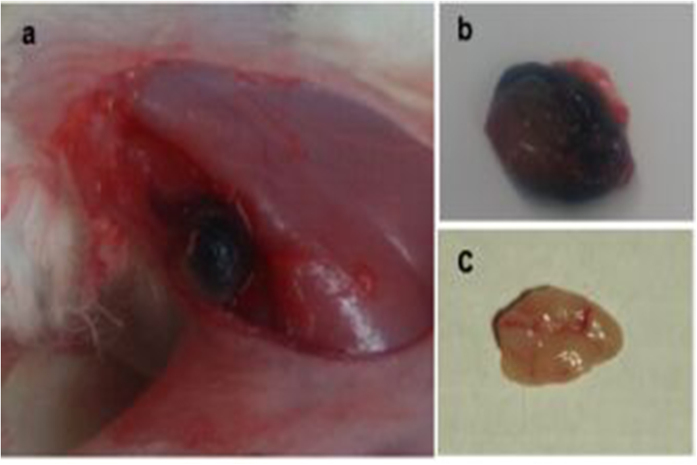
(**a**), the popliteal fossa lymph node in rabbit as stained black. (**b**), the removed dyed LN. (**c)**, the LN injected with saline, exhibiting no signs of staining.

One hour after the injection of CNPs, the rabbits were anesthetized by intravenous injection with 3% pentobarbital solution (1 mL/kg). By gross examination with the naked eye, it was apparent that the popliteal LNs with CNPs injection were stained black (the color of CNs) (Fig. 7a,b), while the LNs with saline injection as control were not stained (Fig. 7c).

### Therapeutic effect of CNPs against tumor

**Figure 8:**
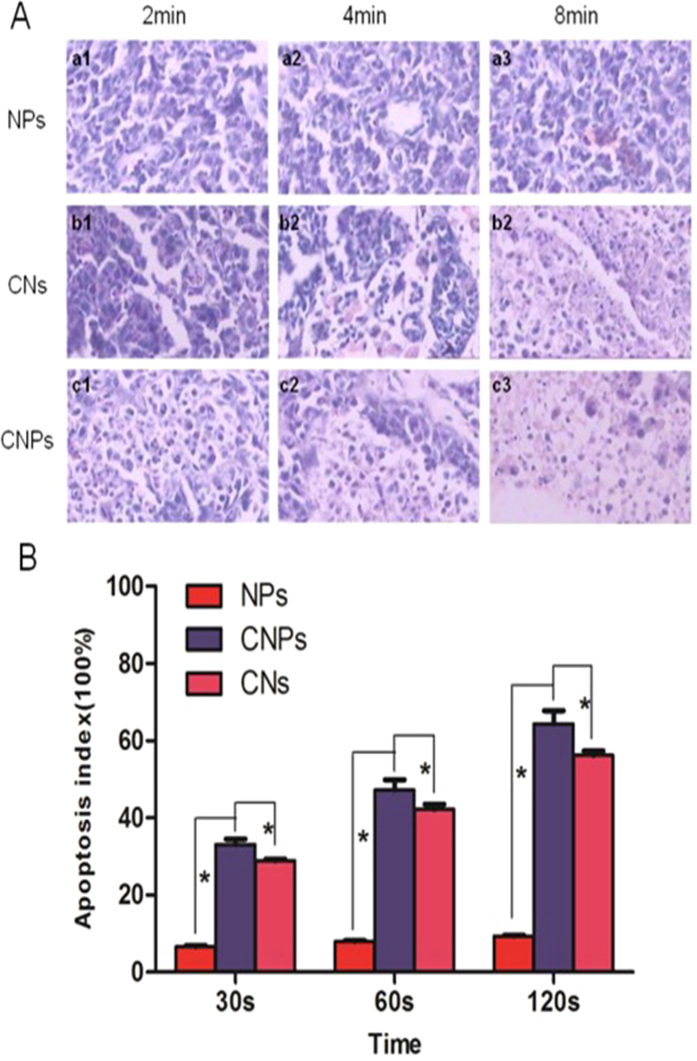
(**A)**, HE staining of metastatic LN tissues after laser exposure. a1–3, injection with NPs after irradiation for 2, 4, 8 min. b1–3, injection with CNs after irradiation for 2, 4, 8 min. c1–3, injection with CNPs after irradiation for 2, 4, 8 min. (**B**), the apoptosis index of different groups after laser exposure for 30, 60 and 120 s (*p < 0.05).

We preliminarily introduced CNPs into tumor metastasis LNs for photothermal therapy both *in vitro* and *in vivo*. The apoptosis rate of CNPs group and CNs group were apparently higher than that of NPs group in each time point. With the extension of irradiation time, cell apoptosis rate obviously increased in groups with CNPs and CNs (Fig. 8B). The apoptosis rate of group with CNPs was a bit higher than that of groups with CNs (P < 0.05). The results indicated that the CNPs had the excellent photothermal therapeutic effect *in vitro*, similar to other similar materials^3^,^32^,^33^.

Lymphatic metastasis was found in all groups under hematoxylin and eosin (HE) examination. Metastatic foci was expressed in good condition in group with NPs (Fig. 8Aa1). However, massive areas of cell apoptosis were found in groups with CNPs and CNs. With the extension of irradiation time, the area of cell apoptosis was also enlarged (Fig. 8Ab1), which further confirmed the photothermal therapeutic effect of CNPs. In addition, the rabbit skin was found without redness, necrosis and ulceration till to one week after laser irradiation, which confirmed the safety of the laser energy.

## Discussion

For the first time, we have demonstrated that the CNs, as a lymph node tracer in clinic, have good optical absorption properties. We also have successfully synthesized a new dual-modality contrast agent for US and PA imaging with CNs. CNPs have shown to be a stable, efficient and safe agent without causing the acute inflammatory reaction^36^,^37^,^38^. The new method with CNPs used for mapping SLN has its advantages compared with combination method (blue dye and nuclide) which is commonly used in clinic. It is a new process with real-time and dual-modality imaging to map SLN, meanwhile, SLN was stained. Dual-modality imaging has complementary advantages from each imaging method. PA imaging has its advantage of high resolution and long duration imaging with proper contrast agent, and US imaging has its advantage of convenient, high depth of penetration, real-time, and dynamic imaging^22^,^39^,^40^. Both real-time imaging and evaluation of SLN before operation can help to decrease the false negative rate in patients with SLNB. The side effects of drug and radiation exposure were also avoided. Dyeing improved the visualization of LNs with naked eyes, which could greatly help surgeon to locate and remove LNs. Therefore, detection and dyeing could be synchronously realized with CNPs instead of a staged procedure with the combination method.

The starting point and duration of CNPs were critical for SLN imaging, both of which determined the real-time imaging observation and whether we had enough time to locate SLN. The findings demonstrated that phase-transition of CNPs could be activated *in vivo* and thus enhanced the PA and US imaging of LNs. Moreover, CNPs could continuously accumulate into LNs and provide excellent PA and US contrast to background during quite a long time for location and operation. However, it was remarkable that some factors could influence the time of both PA imaging and CEUS, such as the thickness of subcutaneous tissue, the massage, etc. As for the thickness of subcutaneous tissue, we tried to implant VX2 tumors underneath the second nipples. After enlargement of the axillary lymph node, the CNPs were subcutaneously injected around the nipples. Both enhancement time of PA imaging and CEUS were delayed to several hours later. It was probably due to the thin subcutaneous tissue around nipples of rabbits, which has fewer lymphatic vessels. Hence, the popliteal fossa lymph nodes were selected, which are the SLNs of the shank tumor. Moreover, the foot pads of rabbits have enough thick subcutaneous tissue, which is rich in lymphatic vessels. It was presumed that the time would be significantly shortened if the CNPs were applied to human body. Massaging the injection site caused an increase in the rate of movement of the CNPs within the lymphatic vessels, which was able to strengthen imaging and accelerate developing time. We still noticed that the amount of CNPs may affect the duration of the imaging, i.e., the higher dose of CNPs may cause longer imaging duration.

Moreover, CNPs have been preliminarily investigated and confirmed for photothermal therapy. The CNPs have been confirmed as a multifunctional agent, which have high efficiency in the therapy of lymphatic metastasis. To the breast cancer patients with unresectable metastatic lymph nodes, the traditional method is radiotherapy and chemotherapy, both of which have great side effects. Photothermal therapy of CNPs will be a potential, supplementary therapeutic method with low side effect and precise treatment to the patients with metastatic lymph nodes. In this work, the CNPs and CNs were confirmed to have photothermal effect both *in vitro* and *in vivo*. Moreover, the CNPs could reach around tumor cells, hence, the tumor cells can be directly damaged by the photothermal-conversion effect from CNPs after laser exposure. However, the apoptosis index of group with CNPs was a bit higher than that of groups with CNs, probably due to the blasting induced by liquid-gas phase transition of CNPs^41^. Furthermore, CNs incorporated in CNPs have been confirmed as an efficient chemotherapeutic drug carrier, which have large surface area and strong adsorption ability. A combination of photothermal therapy and local drug-load chemotherapy in the treatment of metastatic lymph nodes will achieve synergistic effect.

In this study, we did not confirm whether the LNs observed were SLNs or not. Also, the research of CNPs in photothermal and drug-load therapy will be further studied in the future investigations. In short, CNPs have the potential to be multifunctional theranostic applications in both SLNs detection and therapy of metastatic LNs.

## Methods

CNs (50 mg/mL) were purchased from Lummy (China). PFH was purchased from Alfa Aesar (U.K.). PLGA (50:50, 12,000 Da MW) was purchased from Daigang (China). Polyvinyl alcohol (PVA 25,000 Da MW) was obtained from Sigma-Aldrich (USA). Methylene chloride (CH_2_Cl_2_) was purchased from Chuandong (China). Cell Counting Kit-8 (CCK-8) was purchased from Dojindo (Japan). Agarose was purchased from Invitrogen (USA). Annexin V-FITC was purchased from BioVision (USA). Deionized water was obtained with the Milli-Q Plus System (Millipore Corporation, USA).

### Synthesis of CNPs

Carbon nanoparticles nanodroplets (CNPs) encapsulating CNs were fabricated by a modified three-step emulsion process. Four solutions were prepared: (1) 2 mL 2.5% w/v PLGA solution of CH_2_Cl_2_, (2) 8 mL 5% w/v PVA aqueous solution, (3) 0.2 mL 50% w/v CNs aqueous solution and (4) 0.2 mL PFH. CH_2_CL_2_ is a kind of strong solvent and volatile liquid, which was used to dissolve PLGA. After the preparation of CNPs by magnetic stirring, CH_2_CL_2_ was volatilized from CNPs. Medical grade PVA is a safe macromolecular organic matter, which is a kind of common security filmogenhas with high biocompatibility. First, 0.2 mL CNs aqueous solution was added to 0.2 mL PFH and emulsified in an ice bath with an ultrasonic probe (SONICS &MATERALS Inc, USA) at 130 W for 1 min. After that, 2 mL 2.5% w/v PLGA solution of CH_2_Cl_2_ was added to the emulsion and emulsified in the ice bath with the ultrasonic probe at 130 W for another 2 min, and then 8 mL 5% w/v PVA aqueous solution was added to the second emulsion and homogenized (FJ300-SH, China) in an ice bath within 2 min for the third emulsion. Next, the emulsion was stirred for 2 h in an ice bath by a magnetic stirrer (HJ-1, Ronghua, China), and then centrifuged (Eppendorf AG, Germany) at 8000 rpm for 5 min at 4 °C. After centrifugation, the supernatant was discarded, and the precipitate was washed by deionized water. The process was still in an ice bath. Both the centrifugation and washing process were repeated for three times. Finally, the washed spheres were diluted in 2.5 mL degassed deionized water to prepare the stock with the concentration of 20 mg/mL and then stored in a centrifuge tube at 4 °C for further use.

### Characterization of CNPs

A UV/VIS/NIR Spectrophotometer (UV3600, Shimadzu, Japan) was used to acquire the optical absorption spectra of the CNPs and the pure CNs. The morphological, structural characterization and size of CNPs were determined by a transmission electron microscope (TEM, JEM-2100F, Japan) and an optical microscope (Olympus CKX41, Canada). The size distribution and zeta potential of CNPs were measured by a Laser Particle Size Analyzer System (Zeta SIZER3000HS, Malvern, USA).

### Cell culture

The mouse macrophages (RAW264.7) and human breast cancer cell (MDA-MB-231) were obtained from the laboratory of Ultrasound Engineering Institute of Chongqing Medical University and cultured using RPMI 1640 with 10% fetal bovine serum at 37 °C with humidified air containing 5% CO_2_.

### Animal models

All experimental protocols were approved by the Institutional Animal Care and Use Committee of Chongqing Medical University. All the experimental operations of animals were carried out in accordance with the protocol approved by the Institutional Animal Care and Use Committee of Chongqing Medical University. All animals were treated according to the guidelines of the Care and Use of Laboratory Animals. New Zealand white rabbits (weight range of 2.5–4.0 kg) were purchased from Animal Center of Chongqing Medical University. Tumor-bearing rabbits with a VX2 tumor in liver were obtained from the laboratory of Ultrasound Engineering Institute of Chongqing Medical University. The rabbits were anesthetized by intravenous injection with 3% pentobarbital solution (1 mL/kg), and the abdomens of the rabbits were depilated with 8% Na_2_S solution. In the supine position, the rabbits were routinely disinfected. Then, the VX2 tumors in the liver were excised and soaked in sterile saline water. Next, the tumor was sheared into small masses of approximately 1.0 mm^3^ under sterile conditions. Subsequently, after the similar anesthesia, depilating and disinfecting, a hole was created reaching to subskin with 12 G syringe needle in the left lateral hind legs of recipient rabbits, then one piece of tumor tissue was implanted into the hole, and the wound was sutured after hemostasis. Tumor-bearing rabbits were used for experiments when popliteal fossa lymph nodes could be palpated about 3 weeks after tumor inoculation.

### Cytotoxicity of CNPs

The cytotoxicity of the CNPs was evaluated by determining the viability of mouse macrophages (RAW264.7) after incubation in media containing CNPs at concentrations of 0.2, 0.4, 0.6, 0.8 and 1.0 mg/mL, respectively. Control experiments were carried out using the complete growth culture media without CNPs. Cell viability testing was performed through the reduction of the CCK-8 reagent. The cells were first seeded into 96-well plates at a density of 5 × 10^3^ cells per well and incubated at 37 °C and 5% CO_2_ for 24 h. Then, the former medium was replaced with new medium containing at varying concentrations, and the cells were incubated at 37 °C and 5% CO_2_ for another 24 h. After that, 150 μL new media and 10 μL CCK-8 solution were added in each well to replace the former medium, and incubated at 37 °C and 5% CO_2_ for 4 h. At last, the medium was removed and the optical absorbance was then measured at 450 nm on a microplate reader (EL × 800 Universal Microplate Reader, BIO-TEK Instrument Inc., USA). The results were expressed as percentages relative to those obtained in the control experiments. The differences of the results obtained from CNPs and the controls were analyzed statistically by analysis of variance.

### *In vitro* phagocytosis study

The fluorescent dye DiI (1,1′-dioctadecyl-3,3,3′,3′- tetramethylindo-carbocyanine perchlorate)-labeled CNPs were also fabricated by adding DiI in PLGA solution of CH_2_Cl_2_ as mentioned above. The mouse macrophages (RAW264.7) were seeded into 6-well plates 24 h before labeling. The former medium was then replaced with a new medium containing the Dil-labeled CNPs at a concentration of 0.1 mg/mL. The cells were cultured for 3 h and then washed with PBS extensively to remove loosely attached and free CNPs in the medium. The cells were observed under the optical microscope and fluorescence microscope.

### *In vivo* phagocytosis and inflammation study

New Zealand white rabbits with detectable popliteal fossa lymph nodes were anesthetized, depilated and fixed in the side-lying position. These rabbits received a percutaneous injection of CNPs solution (2 mL, 20 mg/mL) via foot pad of the hind legs with lidocaine anesthesia. After the injection for 48 h, all of the lymph nodes were excised, sectioned and stained with hematoxylin and eosin (HE) for pathological examination.

### *In vitro* dual-mode imaging of CNPs

PA signals of four CNPs concentrations (5, 10, 15 and 20 mg/mL) in 2% agarose gel phantom were firstly measured to investigate the relationship between concentrations and PA signals using the VEVO LASR PA imaging system (Vevo®LAZR, Visual Sonics, Inc., Canada), and the saline groups were used as control. The PA signals of both groups were quantitatively analyzed. In addition, the laser energy of our PA system (<30mJ) cannot lead to phase transition of CNPs after multiple tests previously, therefore, Laser Diode Controller (ADR-1085, Mid-river, China) and color Doppler ultrasound MyLab 90 (Esaote, Italy) were used for contrast-enhanced ultrasound imaging (CEUS). Briefly, after exposure to the laser (808 nm, 1 W/cm^2^) for 10 s, four CNPs concentrations (5, 10, 15, 20 mg/mL) in 2% agarose gel phantom were observed with B-mode and contrast-mode, and the saline groups were also used as control. After ultrasonic images acquisition, the images of ROI (region of interest) were quantitatively analyzed with “average gray scale” using DFY ultrasound imaging analysis software (invented by the Institution of Ultrasound Imaging of Chongqing Medical University, Chongqing, China).

### *In vivo* dual-mode imaging of CNPs

New Zealand white rabbits with detectable popliteal fossa lymph nodes were anesthetized, depilated and fixed in the side-lying position. The rabbits were divided into three groups (three in each group) randomly, the CNPs solution (2 ml, 20 mg/mL) group, the NPs solution (2 mL, nanodroplets without any CNs, same PLGA concentration as CNPs) group, and the CNs solution (2 ml, same CNs concentration as that incorporated in CNPs) group, the NPs and CNs groups were taken as control. The popliteal fossa lymph nodes of rabbits were observed using the VEVO LASR PA imaging system with B-mode and PA-mode. Next, these rabbits received a percutaneous injection of CNPs solution (2 mL, 20 mg/mL), NPs solution and CNs solution via foot pad of the hind legs with lidocaine anesthesia respectively, and massage for few minutes. After the solution injection and massage, lymph nodes were intermittently observed for 1 h with PA-mode. Subsequently, under the same anesthesia and position, PA-mode observation was continuous to the point of 24 h and 48 h. All of the average PA signals at each point (pre, 15 min, 1 h, 24 h and 48 h) were recorded and quantitatively analyzed to reveal the rule of lymph nodes PA imaging.

Similar to aforementioned procedure, New Zealand white rabbits with detectable popliteal fossa lymph nodes were observed using color Doppler ultrasound MyLab 90 (Esaote, Italy) with B-mode and contrast-mode. The rabbits were divided into three groups randomly, including the CNPs solution group, the NPs solution group, and the CNs solution group. The dose and concentration of every group were the same with the PA imaging test *in vivo*, and the NPs and CNs groups were taken as control. Next, these rabbits received a percutaneous injection of CNPs solution, NPs solution and CNs solution via foot pad of the hind legs with lidocaine anesthesia respectively, and massage for few minutes. After the solution injection (15 min, 30 min, 1 h and 2 h), the lymph nodes area were exposed to the laser (808 nm, 1 W/cm^2^) for 30 s. Then, the lymph nodes were observed using MyLab 90 with B-mode and contrast-mode. The same laser irradiation was required before each observation. After the images were acquired, the images of ROI were quantitatively analyzed with “average gray scale” using DFY ultrasound imaging analysis software.

### *In vitro* tumor-therapeutic effect of CNPs

The therapeutic effect of the CNPs *in vitro* was firstly evaluated by determining apoptosis rates of MDA-MB-231 cells after incubation in media containing CNPs (20 μL, 20 mg/mL). Control experiments were carried out using the NPs solution (20 μL, the same PLGA concentration as CNPs) and CNs solution (20 μL, the same CNs concentration as that incorporated in CNPs). MDA-MB-231 cells were seeded into 6-well plates at a density of 5 × 10^3^ cells per well and incubated at 37 °C and 5% CO_2_ for 24 h. Then, the former medium was replaced with new medium containing CNPs, NPs and CNs, and the cells were incubated at 37 °C and 5% CO_2_ for 12 h. Subsequently, the cells were exposed to the laser (808 nm, 1 W/cm^2^) for 30 s, 60 s, and 120 s. After that, the cells were incubated at 37 °C and 5% CO_2_ for another 12 h. Then, the cells were collected, washed with 4 °C PBS and centrifuged. This procedure was repeated again. The cells were re-suspended with 250 μL binding buffer, and the concentration of the cells was modulated to 1 × 10^6^/mL. The mixture of 100 μL cell suspension, 5 μL Annexin V/FITC and 10 μL PI solution were added into a 5 ml flow tube, and the cells were incubated in dark place at room temperature for 15 min. Then, 400 μL PBS was added in the tube and the apoptosis rates was determined on a Flow Cytometer (FCM, FACSVantage SE, USA).

### *In vivo* tumor-therapeutic effect of CNPs

The results of CNPs-therapeutic study were further evaluated *in vivo* with the same procedure as described above for *in vivo* imaging part, but the exposure time was extended to 2, 4 and 8 min. All of the lymph nodes were excised, sectioned and stained with HE for pathological examination.

### Statistical analysis

One-way analysis of variance was carried out to calculate the differences among each group. Data was presented as mean ± standard deviation. For statistical analysis, each experiment was repeated at least for three times.

## Additional Information

**How to cite this article**: Yang, L. *et al*. Phase-Transition Nanodroplets for Real-Time Photoacoustic/Ultrasound Dual-Modality Imaging and Photothermal Therapy of Sentinel Lymph Node in Breast Cancer. *Sci. Rep.* **7**, 45213; doi: 10.1038/srep45213 (2017).

**Publisher's note:** Springer Nature remains neutral with regard to jurisdictional claims in published maps and institutional affiliations.

## Supplementary Material

Supplementary Figures
