# Supplementary material for: Phase-Transition Nanodroplets for Real-Time Photoacoustic/Ultrasound Dual-Modality Imaging and Photothermal Therapy of Sentinel Lymph Node in Breast Cancer
Source: Sci Rep. 2017 Mar 24;7:45213. doi: 10.1038/srep45213 (PMC5364557; doi:10.1038/srep45213)
Supplement: Supplementary Figures [file srep45213-s1.pdf]

Supporting information for

Phase-Transition Nanodroplets for Real-Time Photoacoustic/Ultrasound Dual-Modality Imaging and Photothermal Therapy of Sentinel Lymph Node in Breast Cancer

Lu Yang[1,2+], Juan Cheng[1+], Yuli Chen[1], Shengjie Yu[3], Fengqiu Liu[1], Yang Sun[1], Yu Chen[4*] and Haitao Ran[1*]

1. Department of Ultrasound, Second Affiliated Hospital of Chongqing Medical University & Chongqing Key Laboratory of Ultrasound Molecular Imaging, Chongqing, 400010, China.

2. Department of Breast, Thyriod, Pancreas Surgery, Second Affiliated Hospital of Chongqing Medical University, Chongqing 400010, China.

3. Department of Urinary Surgery, Second Affiliated Hospital of Chongqing Medical University, Chongqing 400010, China.

4. State Key Laboratory of High Performance Ceramics and Superfine Microstructure, Shanghai Institute of Ceramics, Chinese Academy of Sciences, Shanghai, 200050, P. R. China.

[+]These authors are contributed equally to this study.

[*]These authors are corresponding authors. Correspondence and requests for materials should be addressed to H. R. Ran (Email: 300190@hospital.cqmu.edu.cn), or Y. Chen (Email: chenyu@mail.sic.ac.cn).

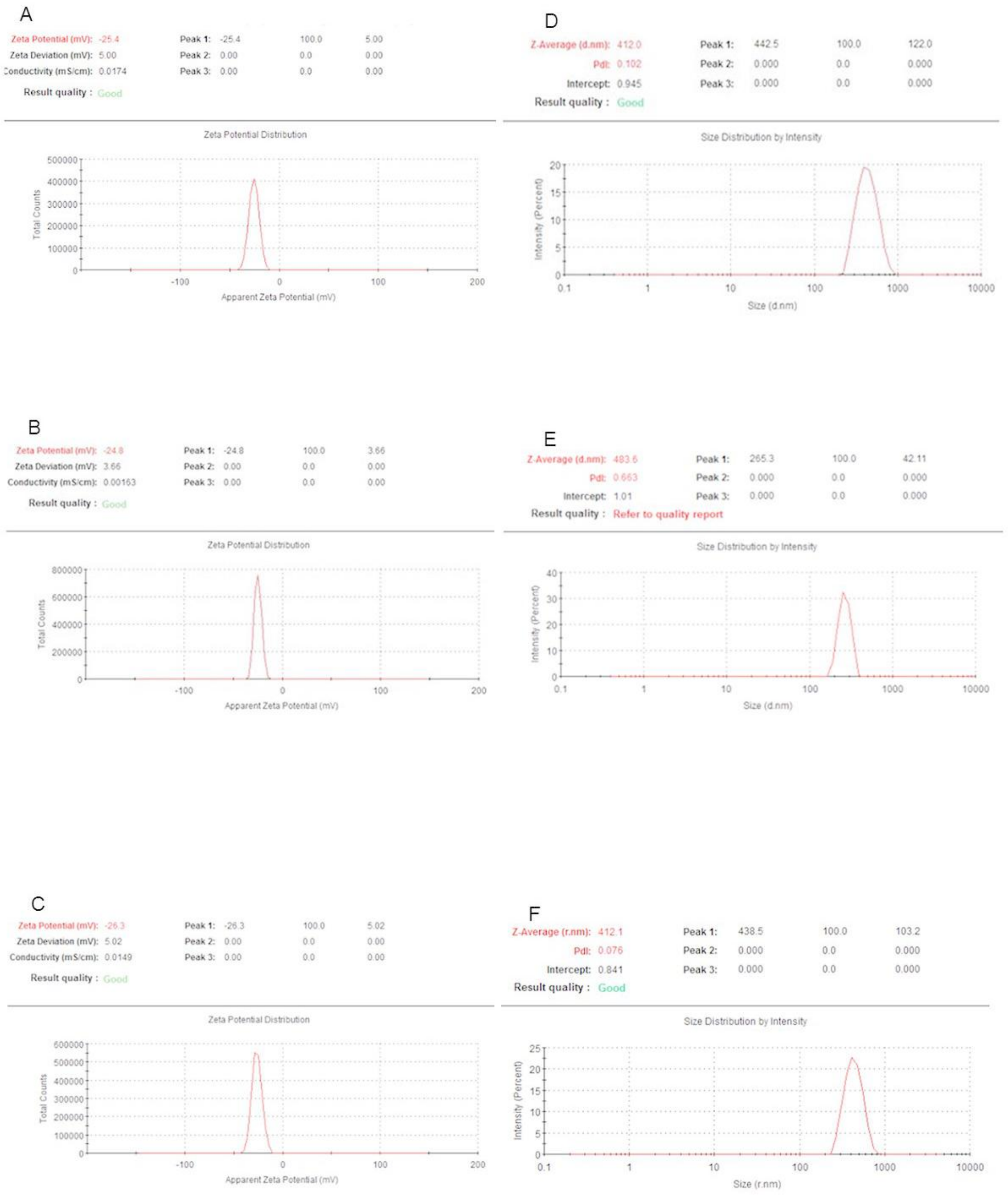

Fig. S1. Zeta potential (A-C) and the size distribution (D-F) of CNPs. The tests of zeta potential and size distribution were conducted for three times.
